# Validation of Diets with Tomato Pomace in Complete Cycle Breeding of *Tenebrio molitor* (L.) (Coleoptera: Tenebrionidae)

**DOI:** 10.3390/insects15040287

**Published:** 2024-04-18

**Authors:** Ferdinando Baldacchino, Anna Spagnoletta, Flutura Lamaj, Maria Luisa Vitale, Vincenzo Verrastro

**Affiliations:** 1 Laboratory of Bioproducts and Bioprocess, ENEA-. Trisaia Research Centre, S.S. Jonica 106, km 419.5, I-75026 Rotondella, Italy; 2 CIHEAM-Bari, Mediterranean Agronomic Institute of Bari, Via Ceglie, 9, I-70100 Valenzano, Italy; lamaj@iamb.it (F.L.); vitale@iamb.it (M.L.V.); verrastro@iamb.it (V.V.)

**Keywords:** yellow mealworm, edible insects, rearing substrate, by-products, yeast, brewer’s grain spent, sterols, linoleic acid, cholesterol

## Abstract

**Simple Summary:**

Yellow mealworm farming (*Tenebrio molitor*, Linnaeus, 1758) for food and feed is considered a more sustainable protein production method than livestock farming. Diets have an impact on the environmental and economic sustainability of the entire production cycle; as a result, there is a great interest in by-product-based diets. However, most evaluations of the efficacy of new diets are generally focused on larval performance, neglecting their suitability for the oviposition phase. The aim of this study was to validate diets supplemented with tomato pomace over a full breeding cycle. As an oviposition substrate, all the tomato pomace-supplemented diets outperformed the bran-yeast control diet (95:5 ratio). During the larval growth phase, the bran-tomato pomace-brewer’s spent grain diet and the bran-tomato pomace-yeast diet performed similarly to the control diet supplemented with yeast. These diets were found to be suitable for the entire production cycle, demonstrating their efficacy in supporting larval growth. In conclusion, tomato pomace can be a valuable by-product in developing effective diets for *T. molitor*, most likely due to its contribution to sterols and fatty acids. Furthermore, its use could provide an alternative to the costly yeast-based supplement.

**Abstract:**

By-product-based diets have the potential to improve the environmental and economic sustainability of *Tenebrio molitor* (Linnaeus, 1758) production. However, evaluations of the efficacy of new diets are generally focused on larval performance, while the effect on adults is poorly understood. This aim of this study was to evaluate diets enriched with tomato pomace over a complete breeding cycle. The results showed that when used as an oviposition substrate, all the tested diets, including tomato pomace (T), outperformed the control bran-yeast diet (WY, 95:5 ratio), possibly due to the presence of cholesterol and linoleic acid. The adults fed with the bran-tomato pomace-brewer’s spent grain diet (WTB, 50:27:23 ratio), the bran-tomato pomace-yeast diet (WTY, 50:41:9 ratio), and the bran-tomato pomace diet (WT, 50:50 ratio) produced significantly more larvae than those fed with the WY diet. The WTB diet (despite being yeast-free) performed similarly to the WY control diet during the subsequent larval growth phase, making it suitable for the entire production cycle. In conclusion, the results show that tomato pomace can be used a valid by-product in the formulation of efficient diets for the breeding of *T. molitor* and also provide an alternative to expensive yeast.

## 1. Introduction

A decade ago, the Food and Agriculture Organization of the United Nations considered rearing insects for feed and food as a potential solution to global protein shortages [1]. The interest in this sector is increasing due to the greater environmental sustainability of insect farming compared with livestock farming [2,3] and the potential application of economic circular principles [4]. However, applying the Life Cycle Assessment (LCA) to black soldier fly rearing has highlighted that the environmental performance of insect-based feed may be beneficial if an appropriate diet is used. Protein diets promote insect growth, but their production has an environmental impact [5]. Mouhrin et al. [6] also identified the consumption of resources for the production of diets as one of the main hotspots of the environmental impact of insect production. The diet also influences the economic sustainability of insect farming; in fact, it was the main variable cost (along with labor) in Dutch mealworm (*Tenebrio molitor* Linnaeus, 1758, Coleoptera: Tenebrionidae) rearing companies [7]. The economic convenience of the diet is determined by its cost and the feed-conversion ratio; this can be calculated using the economic conversion ratio, which is the cost of the diet (€ kg^−1^) required to achieve the increase in larval weight (kg) [8]. This condition stimulates studies on the diets based on by-products, to evaluate their influence on larval performance [9,10].

The diet influences the overall chemical composition and, as well as the nutraceutical quality of the harvested larvae, alters the amino acid profile [11], the fatty acid composition [12,13,14], and the antioxidant content [15]. The ambivalent nature of food bioaccumulation is an important factor to consider when using diets based on by-products and waste from the agri-food industry. In fact, these matrices are a source of valuable phytochemicals with high biological value [16] but could also represent a real risk of the transfer of pesticides and heavy metals to humans [17,18].

Diets can influence mealworms’ immune responses to the entomopathogenic fungi, *Beauveria bassiana*, by increasing gene expression of the antimicrobial peptide, Tenecin 3 [19]. Diets supplemented with the probiotic strains, *Pediococcus pentosacceus* KVL-B19-01 and *Enterococcus faecium* 669, improved *Metarhizium brunneum*-infected growth and larval survival [20].

*T. molitor* is generally raised on a bran-based diet, which is nutritionally unbalanced [21]. The bran is supplemented with protein sources (such as soy protein or yeast) to increase its efficacy [22]. Bran supplemented with yeast is frequently used as a control in studies evaluating the by-products in larval diets [8,23]. However, bran supplemented with yeast (ratio 9:1) has a high economic conversion ratio [8].

The search for cost-effective diets based on a single by-product has frequently revealed high larval mortality and longer growth times [24,25]. Multicomponent diets are more effective at meeting the nutritional requirements of *T. molitor*. To this end, mealworms’ self-selection behavior was also used to determine the proportions of the by-products in the mixtures [23,26,27].

Industrial mealworm farming requires more knowledge for better management [28] and the efficiency of adults.

Diet evaluations are generally carried out on larvae, where an influence on the number of instars and development time has been observed [21], but some authors have demonstrated that diets can influence the survival and reproductive performance of adults [29,30].

The reproduction phase and the production of trays with the appropriate density of larvae are fundamental for rearing efficiency [31,32]. Therefore, since the same diet is used from the oviposition phase, it is important to evaluate the new diets throughout the entire reproductive cycle. Recently, new diets supplemented with tomato pomace were tested on larvae with good results in terms of nutraceutical properties [14]. However, specific evaluations on the influence of such diets on the oviposition phase and early larval development are limited.

This study aims to evaluate the effectiveness of tomato pomace-supplemented diets throughout the entire production cycle.

## 2. Materials and Methods

### 2.1. Diet Compositions

Five diets were formulated using wheat bran supplemented with by-products and yeast. The bran was derived from durum wheat and was purchased from the mill “Molino Cimminelli” (Montegiordano, CS, Italy). The tomato pomace (peels and seeds) was supplied by the organic farm “Antica Enotria” (Cerignola, FG, Italy), as a by-product of the production of tomato puree. The brewer’s spent grain was supplied by a local brewer (Brewery “Jazz Beer”, Bernalda, MT, Italy). The zootechnical dry yeast, as a protein supplement, was purchased from Zabele Srl (Padova, PD, Italy). The by-products were preliminarily dried at 60 °C for 24 h using a Food Dehydrator (COSORI, mod. CP267-FD-RXS, Anaheim, CA 92806, USA). The dried by-products were sieved using a 2 mm manual sieve (Giuliani Tecnologie S.r.l. Torino, TO, Italy), and the coarse part was ground to avoid the influence of particle size [33]. Subsequently, the nutrient composition was determined in a representative sample using AOAC methods [34] (Table 1).

The composition of the five diets, macro-nutrients, and energy values are reported in Table 2. The energy values were calculated using the conversion factors in Regulation (EU) 1169/2011, Annex XIV (Table S1). The wheat bran was used as the first control diet (W) [14], and the supplemented wheat bran yeast (WY) was used as the control rich-protein diet [8]. The other three diets were composed of 50% wheat bran supplemented with tomato pomace and brewer’s spent grain (WTB), tomato pomace and yeast (WTY), and tomato pomace (WT). Diets W, WTB, and WTY were isoprotein formulations; with WT having the lowest protein content.

### 2.2. Insects and Experimental Set-Up

The *T. molitor* colony used in this study was reared at CIHEAM-Bari (Apulia region, Italy) in a climatic room at 28.0 ± 1 °C, 65 ± 5% RH, and 0L:24D photoperiod (except during feeding). The colony was reared on bran and yeast (ratio 95:5 *w*/*w*), supplemented twice a week with pieces of pumpkin as a wet source.

Two experiments (A and B) were conducted at *insectariums* of CIHEAM-Bari for the test diets by imitating a full productive cycle of mass rearing, as described below.

#### 2.2.1. Experiment A

Preliminarily, eight-week-old larvae were fed *ad libitum* with the respective diets to be tested in trays (16.5 cm × 21 cm × 3.5 cm) until pupation. The collected pupae were sexed under a stereoscope (model SMZ745T, Nikon Europe B.V., Amstelveen, The Netherlands), according to the method described by Bhattacharya et al. [35], and stored separately by sex until the beetles’ eclosion.

The experimental design included ten beetles/replicate (five males and five females) and 20 replicates/diet, all assembled using complete randomization. The new adults were placed to lay eggs in plastic cups measuring 13 × 7 cm (h × Ø) containing the same original diet. Every ten days for seven times, the living adults were counted and transferred to new trays with the diet.

According to Berggren et al. [31], the young larvae were counted to estimate adult productivity at one month after oviposition.

#### 2.2.2. Experiment B

To avoid the influence of larval density on their subsequent development [36], groups of 30 young larvae/replicate (6.1 ± 0.1 mg larva^−1^, one month old) from experiment A were fed on the respective diet. The experimental design included ten replicates/diet in a completely randomized design (CRD). For 40 days, larval survival was assessed every ten days on the 300 larvae/diets, and larval weight was measured using an analytical scale (Metter–Toledo, model B2002-S, Milano, Italy; precision ± 0.1 mg). The larval development time was calculated as the number of days between the start of the experiment and the appearance of the first pupa.

Pupae were also sexed, and the first pupae and mature larvae that were collected were weighed. Furthermore, the ingested diet was estimated at the 30th day from the start of the experiment. In each replicate, the weight of the residual diet was subtracted from the initial supplied diet; the residual diet was preliminarily separated from the frass using a sieve (size 0.5 mm).

### 2.3. Statistical Analysis

The data (average value of each replicate) were initially tested for normality, homogeneity, and variance homogeneity. ANOVA was applied to the values, followed by a Tukey–Kramer HDS test post hoc to identify the differences between the diets. The non-parametric Kruskal–Wallis test and pairwise multiple comparisons with Bonferroni correction were applied to the adult survival. The larval survival was assessed by applying a Kaplan–Meier curve analysis on 300 larvae/diets. Significance was assumed at *p* < 0.05. All data were statistically analyzed using the SPSS software version 26.0 (IBM Corporation, Armonk, NY, USA).

## 3. Results

### 3.1. Experiment A

**Figure 1:**
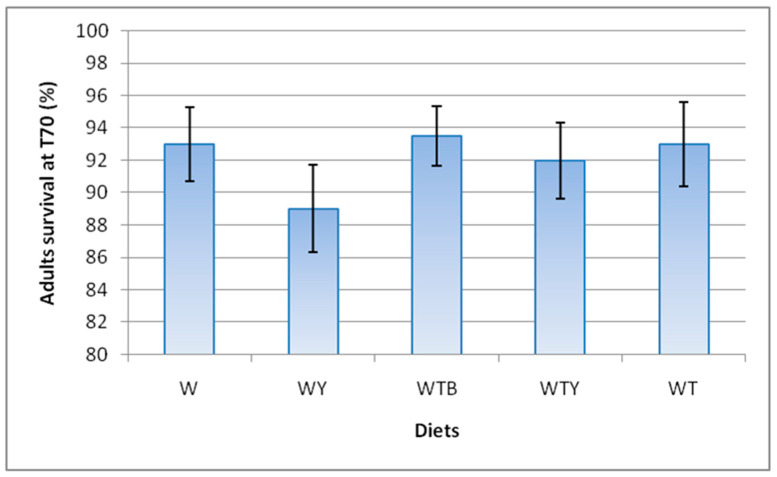
Survival of adults at 70 days from the start of oviposition. Wheat bran (W); wheat bran supplemented with yeast (WY); wheat bran supplemented with tomato pomace and brewer’s spent grain (WTB); wheat bran supplemented with tomato pomace and yeast (WTY); wheat bran supplemented with tomato pomace (WT). The means ± standard error (*n* = 20) were not significantly different at α = 0.05 (Kruskal–Wallis test and pairwise multiple comparisons with Bonferroni correction).

Adult mortality was absent during the first 60 days of the observation. In the last survey (T70), the survival rates ranged from 89.0 ± 2.7% (diet WY) and 93.5 ± 1.8% (diet WTB) (Figure 1). These values were not significantly different between the diets (*H* = 2.161; df = 4; *p* = 0.706), indicating that the tested diets had no effect on the adults’ vitality.

There was a significant difference in larval productivity (mean number of produced larvae) between the oviposition times (two-way ANOVA; *F* = 21.46; df = 6, 665; *p* < 0.0001), between the diets (*F* = 32.04; df = 4, 665; *p* < 0.0001), and with the presence of interaction between the diet and time (*F* = 3.39; df = 24, 665; *p* < 0.0001).

Larval production remained nearly constant for 60 days (Figure S1 reported in the Supplementary Materials) after the oviposition period. During this time, the productivity was significantly higher in the T31–40 interval, and lower in the T41–50 interval. After 60 days of oviposition, the productivity was significantly lower than in the previous period.

**Figure 2:**
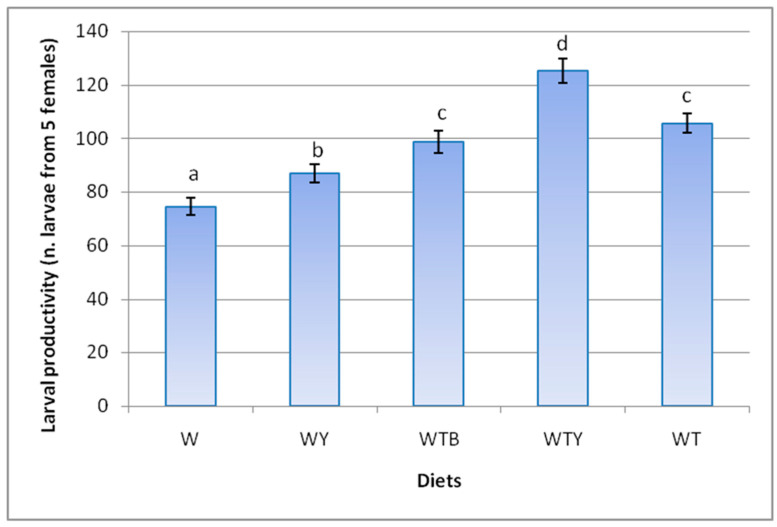
Production of larvae from adults fed on different diets. Wheat bran (W); wheat bran supplemented with yeast (WY); wheat bran supplemented with tomato pomace and brewer’s spent grain (WTB); wheat bran supplemented with tomato pomace and yeast (WTY); wheat bran supplemented with tomato pomace (WT). The means ± SE (*n* = 140) represent the number of larvae produced by five females (replicates) every 10 days during the experimental oviposition period (70 days). The same letters were not significantly different at α = 0.05 (two-way ANOVA and Tukey–Kramer HDS test).

The comparison of the diets highlighted lower average larval production in the control diet (W) based only on bran, whereas productivity was significantly higher in the control diet supplemented with yeast (WY) (Figure 2). Compared with the W and WY control diets, the number of larvae was significantly higher on the WTB diet (+32.5% and +13.4%, respectively) and on the WT diet (+41.9% and +21.4%, respectively). However, the highest larval productivity was obtained on the WTY diet containing yeast. In the latter diet, the number of larvae was 68.1% and 47.7% higher than in the W and WY control diets, respectively.

The number of produced larvae was significantly different between the diets, even at the same oviposition time: T1–10 (*F* = 6.56; df = 4, 95; *p* < 0.0001), T11–20 (*F* = 12.73; df = 4, 95; *p* < 0.0001), T21–30 (*F* = 9.50; df = 4, 95; *p* < 0.0001), T31–40 (*F* = 7.16; df = 4, 95; *p* < 0.0001), T51–60 (*F* = 3.60; df = 4, 95; *p* = 0.009), and T61–70 (*F* = 9.41; df = 4, 95; *p* < 0.0001). Only during the period T41–50 were there no differences between the diets (*F* = 2.16; df = 4, 95; *p* = 0.080). The adults produced significantly more larvae on the WTY diet than on the two control diets (W and WY), from the beginning and for 40 days (up to T31–40) (Table 3). The larvae were always more numerous in the diet supplemented with tomato pomace and brewer’s spent grain (WTB) than in the W control, but were significantly greater only in the T11–20 period. These results are similar to the control supplemented with yeast (WY), with the exception of a significantly lower larval presence on the WTB diet during the T61–70 period. The diet supplemented with tomato pomace only (WT) also produced values that were significantly higher than the W control (periods T11–20, T21–30, and T61–70) and the WY control (only in T11–20) (Table 3).

### 3.2. Experiment B

**Figure 3:**
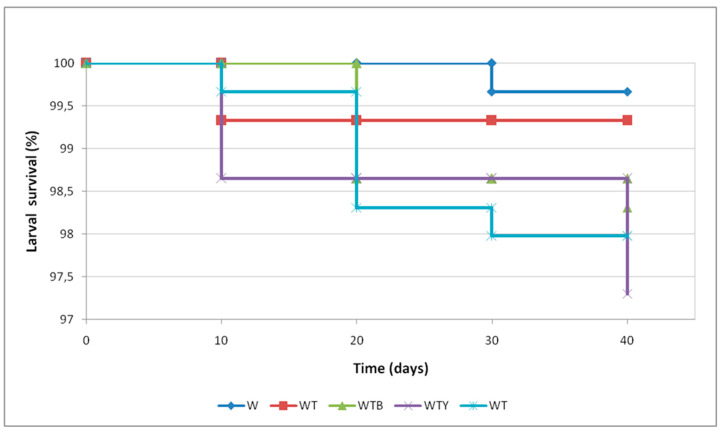
Larval survival at different growth times on the different tested diets. Wheat bran (W); wheat bran supplemented with yeast (WY); wheat bran supplemented with tomato pomace and brewer’s spent grain (WTB); wheat bran supplemented with tomato pomace and yeast (WTY); wheat bran supplemented with tomato pomace (WT). Kaplan–Meier analysis and log–rank test (*p* < 0.05).

Larval survival was between 99.7% and 97.3% at the end of the observation period. The Kaplan–Meier survival curve for larvae was not significantly different between the diets (*p* = 0.106) (Figure 3).

**Figure 4:**
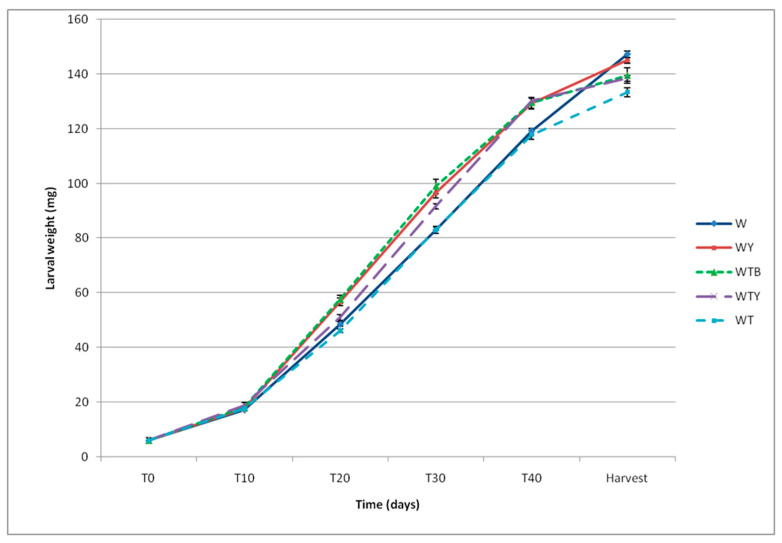
Individual larval weight depends on diet and development time (days) from the start of the experiment. Wheat bran (W); wheat bran supplemented with yeast (WY); wheat bran supplemented with tomato pomace and brewer’s spent grain (WTB); wheat bran supplemented with tomato pomace and yeast (WTY); wheat bran supplemented with tomato pomace (WT). The means ± SE (*n* = 10) with the same letter in the same column were not significantly different at α = 0.05 (one-way ANOVA followed by Tukey–Kramer HSD test).

The weight increase of the larvae during the observation period is reported in Figure 4. The analysis of the average weight of the young larvae showed no statistically significant differences between the diets, both at the start of the experiment (T0) (*F* = 1.32; df = 4, 45; *p* = 0.279) and after 10 days of growth (T10) (*F* = 2.02; df = 4, 45; *p* = 0.108) (Table S2 reported in the Supplementary Materials). The influence of diet on larval weight was highlighted at T20 (*F* = 15.20; df = 4, 45; *p* < 0.0001); in fact, the larvae fed on the WY and WTB diets weighed significantly more than the other larvae. The W and WTY diets provided the same results, while the WT diet was not significantly different from the W diet (wheat bran without yeast). At T30 (*F* = 13.77; df = 4, 45; *p* < 0.0001) and T40 (*F* = 8.448; df = 4, 45; *p* < 0.0001), the larvae fed on the WY, WTB, and WTY diets gained significantly greater weights than those fed on the W and WT diets but did not differ significantly from each other. The weight of the larvae at harvest was significantly different between the diets (*F* = 9.076; df = 4, 45; *p* < 0.0001), but with larger values on the W and WY diets; the WTB and WTY diets were found to be no different from the yeast-supplemented control (WY) and the WT diet (the latter reporting lower larval weights).

The effects of the tested diets on the larval development time (from the start of the experiment to the presence of the first pupa) were significantly different (*H* = 20.723; df = 4, *p* < 0.0001), as shown in Table 4. The largest number of days (45.1 days) was recorded on the W control diet, with the values not significantly different from the WTB and WT diets; the lowest number of days (39.5 days) was obtained on the WTY diet, with the values not significantly different from the WY and WTB diets. This allowed us to obtain an advance in pupation of 4.9 and 5.6 days for the WY and WTY diets, respectively, compared with the wheat bran-only control (W).

The analysis of the first pupa weight did not reveal significant differences between the diets (*F* = 1.561; df = 45, 4; *p* = 0.201), with the values ranging from 116.1 to 125.0 mg (Table 4). All the diets had no influence on the male:female ratio (50/50); this ratio was not significantly different even in the WTY diet, which favored females (42/58). The diet ingestion was significantly different between the diets but did not appear to be exclusively linked to larval weight or development time. This was best shown by the W and WT diets (which were low in nutrients), which were found to be comparable in terms of the development rate and the larval weight up to T30. Therefore, we hypothesize the intervention of other dietary factors that were not considered in this investigation. 

## 4. Discussion

Despite extensive scientific research on the use of by-products in diet formulation, there is still a significant lack of evaluations of the same diets in *T. molitor* adults. Since a single diet is used from the oviposition phase, understanding the impact of these diets is crucial for breeding efficiency. Our findings fill a gap for some previously tested diets on larvae [14] by assessing their impact across the entire breeding cycle.

Diet can negatively affect adult survival when using chicken feed [30]. 

According to the findings of Morales-Ramos et al. [37], none of the diets we tested had a negative impact on adult survival in the first two months. Mortality began only after 61–70 days of oviposition, with survival rates still high (>89%). From this point of view, the tested diets were all suitable, since it is suggested that the beetles are replaced between the 58th and 74th day to obtain the maximum oviposition efficiency [37]. However, this criterion was based on the mortality and oviposition curve per female, which, in our case, was much more constant (up to the 60th day) compared with the progressive decline from the third week detected by Morales-Ramos et al. [37]. As a result, adult replacement could be reconsidered in light of the oviposition curves determined by the various diets.

It has been found that adult productivity can be significantly improved by consuming diets containing tomato pomace. In fact, the productivity levels were much higher than those of the control group, which included both bran and bran supplemented with yeast. It is worth noting that this improvement cannot be attributed to the positive effects of proteins on fertility [26,38]. The diets that were tested had similar protein levels to the bran-only control (16.7%) but less protein than the yeast-supplemented bran control (18.2%).

Interestingly, the diet that consisted of a 50:50 ratio of bran and tomato pomace and had the lowest protein content (13.1%) actually recorded higher reproduction rates than the higher protein control. This suggests that the tomato pomace has properties that contribute to improved productivity and cannot be solely attributed to protein content.

The reproductive advantage recorded in all the diets supplemented with tomato pomace could be attributed mainly to cholesterol, which is present in tomato seeds quantitatively secondary to β-sitosterol [39,40]. Insects rely on phytosterols from plants and fungi since they cannot produce endogenous sterols [41]. Cholesterol is more efficiently utilized by insects than the phytosterols, sitosterol and ergosterol, although with large differences between species [42]. Sterols play a crucial role in insect growth, affecting both tissue structure and function. Cholesterol, specifically, is vital for insect fertility since it is necessary for the synthesis of the hormones involved in growth, molting, and ecdyson [42]. Research shows that a cholesterol-deficient diet can negatively affect the egg viability in *Musca domestica* L. However, it does not seem to affect adult survival or the number of eggs laid [43]. Our findings align with studies that have evaluated bran-based supplements, which showed that cholesterol enrichment can lead to more female progeny [26]. While these studies considered cholesterol supplementation expensive, our use of tomato pomace as a by-product provides a cost-effective source of cholesterol.

Tomato pomace, besides being a source of sterols, also contains “essential” polyunsaturated fatty acids (PUFAs), such as ω-6 linoleic acid (LA) and ω-3 linolenic acid (ALA) [44,45]. These PUFAs may have contributed to positively modulating the fertility of *T. molitor* adults.

Dietary ω-3 and ω-6 supplementation improved fertility in humans and animals [46,47,48] by improving the quality and fluidity of the seminal fluid membranes [49].

Polyunsaturated fatty acids (PUFAs) play a crucial role in the biosynthesis of arachidonic acid (AA) and indirectly participate in the production of prostaglandin E_2_ (PGE_2_) [50,51]. PGE_2_ has a significant impact on various physiological processes such as fluid secretion, immunity, ageing, and reproduction in insects, similar to vertebrates [52]. For instance, PGE_2_ stimulates egg-laying behavior in insects such as *Acheta domesticus* and *Teleogryllus commodus* [53,54,55]. Additionally, PGE_2_ plays a crucial role in egg development in *Rhodnius prolixus* [56]. The levels of PGE_2_ are usually high in the spermatheca of non-virgin and mated females, and its synthesis is facilitated by enzymes that are transferred through spermatophores [57]. The lipid content of the spermatophore is essential in egg production and hormone synthesis and is sensitive to changes in the nutritional and health status of males [58].

Studies on the fatty acid composition of some tissues in *T. molitor* adults revealed high levels of radioactive AA accumulation at the testicular level [59]. *Dysdercus cingulatus* also showed an increase in phospholipids in body fat and gonadal tissues, which was associated with adult maturation [60].

In mammals, PGE_2_ biosynthesis starts with the hydrolysis of AA from the cellular phospholipids by phospholipase A_2_ (PLA_2_) [61].

Most insects lack or contain trace amounts of C20 PUFAs, such as AA [62,63]. To compensate for the lack of AA, insects have developed a new PGE_2_ biosynthetic mechanism [64]. Insect PLA_2_ catalyzes the release of C18 PUFAs (such as LA and ALA) from membrane phospholipids, which are then converted to AA via mammalian-like elongation/desaturation pathways [55,64]. The enzymes involved in this process include ∆6 desaturase and elongase [57]. These PUFAs are known to have a high affinity and modulating capacity for these enzymes [48].

In a previous study, it was found that diets supplemented with tomato pomace resulted in a significant increase in the lipid fraction of *T. molitor* larvae, including LA, ALA, and the ω-3/ω-6 ratio, compared with the standard diet [14]. This led us to hypothesize that these PUFAs introduced with the diet and accumulated during the larval period have contributed to improving the reproductive performances of adults. LA and ALA are important precursors of the biosynthesis of the reproductive eicosanoid, PGE_2_, and are also major components of body fat. Therefore, their increased presence in the larvae may play a significant role in enhancing the reproductive performance of the adult insects.

Our tested diets also satisfied the need for sterols by *T. molitor* larvae, whose growth is favored by the presence in the diet of cholesterol, sitosterol, stigmasterol, stigmastanol, and ergosterol [42]. In the mealworm diets, sterols are provided via vegetables and yeast [65].

Vegetables are a good source of phytosterols, such as β-sitosterol, campesterol, stigmasterol, β-sitostanol, and campestanol, which vary in amounts depending on the species [66]. Meanwhile, ergosterol can be found in yeast [67]. It is worth noting that the brewer’s spent grain contains sterols such as cholesterol, ergosterol, brassicasterol, campesterol, campestanol, stigmasterol, and β-sitosterol [68], but the study by Rio et al. [69] did not mention cholesterol as one of the sterols they found.

All the tested diets contained sterols, but they affected larval growth differently. The control diet, consisting of bran only, and the bran-tomato diet (WT) showcased similar larval weights and development times during the growth period except for at harvest. A lower weight of the larvae at harvest on the WT diet can be attributed to its lower protein content, which was not immediately evident, perhaps due to the lower protein requirement of the young larvae [23]. The control diet that was supplemented with yeast yielded the best results, which was consistent with other authors’ findings [30]. However, the other two diets containing tomato pomace (WTB and WTY) yielded similar results in terms of the development times and larval growth throughout the developmental period and at harvest. These last two diets, which have already been tested in previous work, had given higher larval weights but were not significantly different from the bran-only control [14]. In this study, administering such diets from the first phase of larval growth could be the basis of significant improvement.

The bran-tomato pomace-brewer’s spent grain (WTB) diet is a less expensive alternative to the bran diet supplemented with yeast since it does not contain yeast. This makes it a more economical solution for the entire production cycle. Conversely, the WT diet was not as efficient as the yeast-supplemented bran for subsequent larval growth even though it was better in the oviposition phase. Therefore, the adults’ performance was significantly amplified by all the diets supplemented with tomato pomace. Testing the diets in both production phases can enhance mealworm farming efficiency.

## 5. Conclusions

The impact of diets on larval performance, nutritional composition of the collected larvae, and production costs are essential factors for the economic and environmental sustainability of *T. molitor* farming. The outcomes of this study indicate that all tested diets, including tomato pomace, were better than the bran-yeast control diet when utilized as an oviposition substrate. This could be due to the contribution of cholesterol and linoleic acid. The bran-tomato pomace-brewer’s spent grain diet can be the only diet for the entire production cycle. These results are a step forward towards achieving greater efficiency and economic sustainability in *T. molitor* farming.

## Figures and Tables

**Table 1:** Nutrient composition of the ingredients preliminarily conditioned (% DM).

Ingredient	Dry Matter (%)	Crude Protein (%)	Crude Fat (%)	Crude Fiber (%)	Ash (%)	Carbohydrate (%)
Wheat bran	91.2	16.7	6.5	36.1	4.2	30.2
Tomato pomace	92.1	9.5	3.2	67.1	3.9	8.9
Brewer’s spent grain	93.4	24.7	4.8	42.0	2.6	24.0
Yeast	93.0	47.6	2.4	6.8	8.0	13.8

**Table 2:** Diet compositions and nutritional values * (% DM).

Diet	Wheat Bran (%)	Tomato Pomace (%)	Brewer’s Spent Grain (%)	Yeast (%)	Protein Value (%)	Carbohydrate (%)	Crude Fiber (%)	Fat (%)	Energy (kcal/100 g)
W	100	-	-	-	16.7	30.2	36.1	6.5	318.2
WY	95	-	-	5	18.2	29.4	34.6	6.3	316.5
WTB	50	27	23	-	16.6	23.0	45.8	5.2	297.1
WTY	50	41	-	9	16.5	20.0	46.2	4.8	281.4
WT	50	50	-	-	13.1	19.6	51.6	4.9	277.5

* Calculated values; wheat bran (W); wheat bran supplemented with yeast (WY); wheat bran supplemented with tomato pomace and brewer’s spent grain (WTB); wheat bran supplemented with tomato pomace and yeast (WTY); wheat bran supplemented with tomato pomace (WT).

**Table 3:** Production of larvae on different diets in different oviposition times.

Diets	T1–10	T11–20	T21–30	T31–40	T41–50	T51–60	T61–70
W	85.6 ± 13.7 ^a^	59.4 ± 4.0 ^a^	57.7 ± 5.8 ^a^	100.2 ± 6.5 ^a^	83.7 ± 7.1 ^a^	94.6 ± 5.1 ^ab^	41.0 ± 3.0 ^a^
WY	71.5 ± 10.3 ^a^	74.4 ± 7.0 ^ab^	104.6 ± 10.8 ^bc^	95.7 ± 10.8 ^a^	86.7 ± 7.9 ^a^	107.4 ± 6.1 ^ab^	69.6 ± 5.6 ^b^
WTB	114.9 ± 9.7 ^ab^	98.5 ± 10.7 ^bc^	95.4 ± 10.7 ^ab^	128.2 ± 11.0 ^ab^	86.0 ± 6.4 ^a^	121.8 ± 9.2 ^ab^	46.4 ± 53 ^a^
WTY	151.6 ± 13.0 ^b^	140.4 ± 12.3 ^d^	140.5 ± 13.0 ^c^	157.9 ± 9.9 ^b^	88.5 ± 7.4 ^a^	125.8 ± 8.2 ^b^	71.8 ± 7.5 ^b^
WT	97.8 ± 13.2 ^a^	112.7 ± 8.3 ^cd^	117.1 ± 7.8 ^bc^	127.6 ± 8.0 ^ab^	109.2 ± 6.6 ^a^	92.2 ± 10.4 ^a^	83.3 ± 6.9 ^b^

Wheat bran (W); wheat bran supplemented with yeast (WY); wheat bran supplemented with tomato pomace and brewer’s spent grain (WTB); wheat bran supplemented with tomato pomace and yeast (WTY); wheat bran supplemented with tomato pomace (WT). The means ± SE *(n* = 20) indicate the number of larvae produced by five females (replicates) in 10 days. The same letters within the same oviposition period were not significantly different at α = 0.05 (one-way ANOVA and Tukey–Kramer HDS test).

**Table 4:** Effects of diets on early pupation, pupal weight, sex ratio, and ingested diet.

Diets	Larval Development Time (Days)	Early Pupation ^1^ (Days)	Weight Pupae (mg)	Sex Ratio (Male:Female)	Ingested Diet at T30 (g/replicate)
W	45.1 ± 0.7 ^b^	0	116.1 ± 3.0 ^a^	50/50	9.1 ± 0.2 ^c^
WY	40.2 ± 0.7 ^a^	−4.9	116.5 ± 3.2 ^a^	47/53	8.9 ± 0.2 ^c^
WTB	42.3 ± 0.9 ^ab^	−2.8	125.0 ± 2.7 ^a^	51/49	10.0 ± 0.2 ^d^
WTY	39.5 ± 1.1 ^a^	−5.6	121.7 ± 3.8 ^a^	42/58	7.9 ± 0.2 ^b^
WT	43.9 ± 0.9 ^b^	−1.2	122.6 ± 2.9 ^a^	46/54	7.1 ± 0.2 ^a^

^1^ n. days compared with the wheat bran-only control (W). Wheat bran (W); wheat bran supplemented with yeast (WY); wheat bran supplemented with tomato pomace and brewer’s spent grain (WTB); wheat bran supplemented with tomato pomace and yeast (WTY); wheat bran supplemented with tomato pomace (WT). The means ± SE (*n* = 10) with the same letter in the same column were not significantly different at α = 0.05 (larval development time: Kruskal–Wallis test and pairwise multiple comparisons with Bonferroni correction; weight pupae and ingested diet: one-way ANOVA followed by Tukey–Kramer HSD test; sex ratio: χ2 test).

## Data Availability

All data from this experiment are contained in the article.

## Supplementary Materials

The following supporting information can be downloaded at https://www.mdpi.com/article/10.3390/insects15040287/s1: Table S1: Conversion factors for the calculation of energy; Figure S1: Production of larvae from adults at different oviposition periods. The means ± SE (*n* = 100) represent the number of larvae produced by five females (replicate) every 10 days during the experimental oviposition period (70 days). The values with the same letter were not significantly different at α = 0.05 (two-way ANOVA and Tukey–Kramer HDS test); Table S2: Individual larval weight depends on diet and development time (days) from the start of the experiment. Wheat bran (W); wheat bran supplemented with yeast (WY); wheat bran supplemented with tomato pomace and brewer’s spent grain (WTB); wheat bran supplemented with tomato pomace and yeast (WTY); wheat bran supplemented with tomato pomace (WT). The means ± SE (*n* = 10) with the same letter in the same column were not significantly different at α = 0.05 (one-way ANOVA followed by Tukey–Kramer HSD test).
