# Supplementary material for: Validation of Diets with Tomato Pomace in Complete Cycle Breeding of Tenebrio molitor (L.) (Coleoptera: Tenebrionidae)
Source: Insects. 2024 Apr 18;15(4):287. doi: 10.3390/insects15040287 (PMC11050266; doi:10.3390/insects15040287)
Supplement: Supplementary file 1 [file insects-15-00287-s001.zip › insects-2927785-supplementary.pdf]

Supplementary Materials

Table S1. Conversion factors for the calculation of energy (source: Regulation (EU) 1169/2011, Annex XIV).

Nutrient	Conversion Factors
Carbohydrate (except polyols)	17 kJ/g - 4 kcal/g
Polyols	10 kJ/g - 2,4 kcal/g
Protein	17 kJ/g - 4 kcal/g
Fat	37 kJ/g - 9 kcal/g
Salatrims	25 kJ/g - 6 kcal/g
Alcohol (ethanol)	29 kJ/g - 7 kcal/g
Organic acid	13 kJ/g - 3 kcal/g
Fibr	8 kJ/g - 2 kcal/g
Erythritol	0 kJ/g - 0 kcal/g

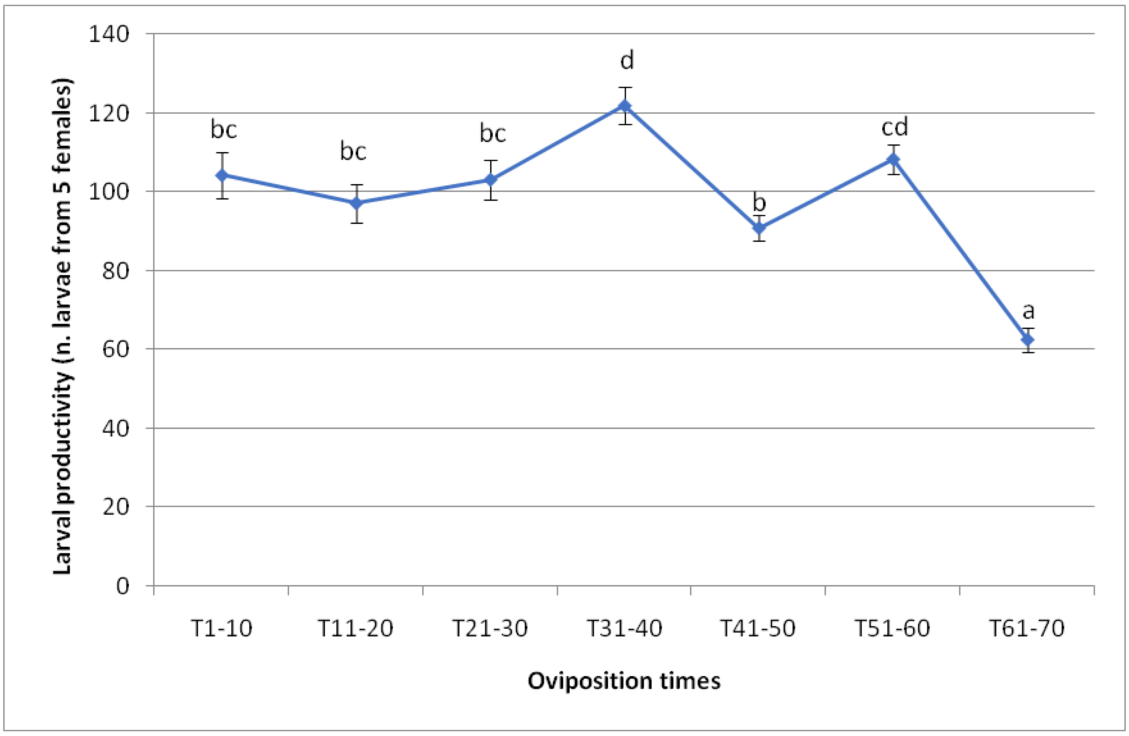

Figure S1. Production of larvae from adults at different oviposition periods. The mean ± SE (n = 100) represents the number of larvae produced by five females (replicates) every10 days during the experimental oviposition period (70 days). Values with the same letter are not significantly different at $\alpha = 0.05$ (two-way ANOVA and Tukey-Kramer HDS test).

Table S2. Individual larval weight depends on diet and development time (days) from the start of the experiment.

Diets	T0 (mg larvae^{-1})	T10 (mg larvae^{-1})	T20 (mg larvae^{-1})	T30 (mg larvae^{-1})	T40 (mg larvae^{-1})	Harvest (mg larvae^{-1})
W	6.1±0.1 ^{a}	17.2±0.4 ^{a}	48.6±0.9 ^{ab}	83.0±1.2 ^{a}	119.2±0.9^{a}	147.2±1.2 ^{c}
WY	6.1±0.0^{a}	17.5±0.4 ^{a}	56.7±1.1 ^{c}	96.4±1.7 ^{b}	129.2±2.0^{b}	145.0±1.0 ^{bc}
WTB	6.1±0.1 ^{a}	18.0±0.4 ^{a}	57.7±1.4 ^{c}	98.9±2.7 ^{b}	129.5±2.1^{b}	139.4±2.8 ^{ab}
WTY	6.2±0.0 ^{a}	18.9±0.5 ^{a}	51.0±1.8 ^{b}	91.7±2.6 ^{b}	130.1±3.3^{b}	138.4±2.0 ^{ab}
WT	6.0±0.0 ^{a}	17.7±0.6 ^{a}	46.2±0.8 ^{a}	83.0±1.3 ^{a}	117.8±1.7^{a}	133.3±1.7 ^{a}

Wheat bran (W); wheat bran supplemented whit yeast (WY); wheat bran supplemented with tomato pomace and brewer's spent grain (WTB); wheat bran supplemented whit tomato pomace and yeast (WTY); wheat bran supplemented whit tomato pomace (WT). Means ± SE (n = 10) with the same letter in the same column are not significantly different at α = 0.05 (one-way ANOVA followed by Tukey-Kramer HSD test).
